# Reliability and validity of the Thai version of the Rapid Mood Screener (RMS-T)

**DOI:** 10.1371/journal.pone.0355733

**Published:** 2026-08-06

**Authors:** Chayanis Som-On, Chawisa Suradom, Roger S. McIntyre, Sirijit Suttajit, Suttipong Kawilapat, Sutrak Pilakanta, Manit Srisurapanont

**Affiliations:** 1 Department of Psychiatry, Faculty of Medicine, Chiang Mai University, Chiang Mai, Thailand; 2 Department of Psychiatry, University of Toronto, Ontario, Canada; 3 Department of Pharmacology and Toxicology, University of Toronto, Ontario, Canada; 4 Department of Statistics, Faculty of Science, Chiang Mai University, Chiang Mai, Thailand; Jawaharlal Institute of Postgraduate Medical Education and Research, INDIA

## Abstract

**Purpose:**

Bipolar disorder (BD) is frequently misdiagnosed as unipolar depression, leading to inappropriate treatment and adverse outcomes. The Rapid Mood Screener (RMS) was developed to improve BD detection, a prerequisite for improving health outcomes in BD. This study aimed to translate and culturally adapt the RMS into Thai (RMS-T) and to evaluate its psychometric properties in Thai adults with bipolar I disorder (BD-I) and bipolar II disorder (BD-II).

**Methods:**

The RMS was translated using forward–backward translation and cultural adaptation. Participants included outpatients at a tertiary psychiatric clinic with diagnosed major depressive disorder (MDD; n = 109) and BD (n = 31; BD-I = 21, BD-II = 10). Psychometric properties included internal consistency, item-total correlations, test–retest reliability, and criterion validity against the Mini International Neuropsychiatric Interview (MINI-5). Receiver operating characteristic (ROC) analyses compared RMS-T with the Thai Mood Disorder Questionnaire (T-MDQ), and optimal cutoffs were determined using Youden’s index.

**Results:**

Internal consistency was acceptable for a brief six-item screening tool (Cronbach’s alpha = 0.610; McDonald’s Omega = 0.632). RMS-T demonstrated good discriminative ability for BD (AUC = 0.805), comparable to T-MDQ. Optimal cutoffs were ≥ 4 for BD (sensitivity 64.5%, specificity 78.9%, Youden’s index 0.43). Test–retest reliability was strong.

**Conclusion:**

The RMS-T is a valid, practical, and time-efficient screening tool for BD patients presenting with depressive episodes. Further research should investigate end-user satisfaction, the impact on health outcomes, clinical utility, and cost-effectiveness in broader settings and populations.

## Introduction

Bipolar disorder (BD) is a severe psychiatric condition affecting approximately 1–3% of the population [1,2]. Diagnosis is often delayed, with an average gap of six years between symptom onset and initiation of appropriate treatment [3,4]. Misdiagnosis as unipolar depression occurs in up to 60% of cases, and nearly one-quarter of patients treated for major depressive disorder (MDD) actually have BD [4]. Only about 20% of individuals experiencing a depressive episode receive an accurate BD diagnosis within the first year [5], as depressive episodes frequently constitute the initial presentation, and the transition to BD typically occurs later [6]. In many instances, this misdiagnosis or delayed recognition is inadvertent insofar as depressive episodes are often the index presentation, and the transition to BD often occurs subsequently. Such delays are associated with poorer functional outcomes, increased suicide risk [7–9], and reduced responsiveness to mood-stabilizing medications [10]. Later transition to BD is also linked to worse baseline psychosocial functioning, underscoring the clinical consequences of delayed recognition [6,11].

Several self-report screening instruments for BD exist, including the 32-item Hypomania Checklist (HCL-32), Bipolar Spectrum Diagnostic Scale (BSDS), and the Mood Disorder Questionnaire (MDQ). However, their use in routine clinical practice is limited by time constraints, uncertainty about when to screen, and conceptual concerns regarding their clinical value [12,13]. A nationwide electronic survey reported that only 32% of 200 healthcare practitioners (HCPs) used a BD screening tool, and only 29% currently use the MDQ, despite 85% being familiar with it [14]. Across studies, the MDQ demonstrated moderate sensitivity (61.3%) and high specificity (87.5%), with better detection of BD-I than BD-II (66.3% vs. 38.6%). Lowering the cutoff improves sensitivity but compromises specificity, and findings on optimal thresholds remain inconsistent [15]. In addition, no studies have simultaneously examined both the potential benefits of improved detection against the risks and costs of overdiagnosis. Consequently, while MDQ and similar tools may aid detection, routine clinical use cannot be recommended based on current evidence, underscoring the need for further research on their cost-effectiveness and diagnostic impact.

The Rapid Mood Screener (RMS) is a recently developed, self-administered screening tool designed to differentiate bipolar I disorder (BD-I) from MDD in patients with depressive symptoms, which contains six items based on manic symptoms and bipolar depression risk factors. It demonstrated high sensitivity (0.88) and specificity (0.80) when four or more items were endorsed. The RMS can be completed in under two minutes during or outside of a clinical visit (e.g., online, via electronic medical record system, waiting room), making the RMS a patient-friendly screener that can be easily integrated into clinical practice, with most HCPs (81%) reporting a preference for RMS over MDQ [14]. Over two-thirds of respondents thought that the RMS was better than other screening instruments, and 84% believed that the new RMS would have a positive impact on their practice, while 76% were likely to screen new patients with depressive symptoms using the RMS [14,16]. Cost analysis indicates potential economic savings when routinely administering a screening tool such as the RMS [17].

Validation studies have been conducted for RMS in the United States and China, including assessments in Bipolar II disorder (BD-II) [18]. Unfortunately, no culturally adapted version exists for Thai or any other Southeast Asian clinical settings, leaving the Thai version of the MDQ the sole yet still not widely used option for bipolar screening in Thailand. Therefore, this study aimed to translate the RMS into Thai (RMS-T) and evaluate its reliability and validity in Thai adults with BD-I and BD-II in psychiatric outpatient settings. Although recent literature suggests that routine use of the MDQ is limited by moderate sensitivity and conceptual concerns, the validated MDQ serves as a necessary benchmark for evaluating the convergent validity of the new Thai RMS-T.

## Materials and methods

### Study population

#### Inclusion criteria.

i) Adults aged 20–65 years;
ii) Ability to read, speak, and understand Thai;
iii) Ability to use Google Forms on a smartphone, tablet, or computer; and
iv) Meeting the Diagnostic and Statistical Manual of Mental Disorders, Fifth Edition, Text Revision (DSM-5-TR) and Mini International Neuropsychiatric Interview (MINI) criteria for BD-I/BD-II or MDD.

#### Exclusion criteria.

i) Inability to understand or complete the questionnaire due to educational level, language limitations, or severe psychotic symptoms; and
ii) Other psychiatric, substance use, or physical disorders that limit their ability to provide accurate information.

### Sample size and statistical determination

The required sample size was calculated using Buderer’s formula for diagnostic test studies. Assuming an anticipated sensitivity of 0.88 and specificity of 0.80, a prevalence of BD among MDD patients of 0.22 from a previous study conducted similarly in a psychiatric outpatient unit, the estimated sample size was 140 participants (31 BD, 109 MDD) to achieve adequate precision for diagnostic validation [19,20].

### Recruitment of participants

The study protocol was approved by the Research Ethics Committee of the Faculty of Medicine, Chiang Mai University (Study Code: PSY-2567–0383), and written informed consent was obtained from participants. Participants were recruited from the psychiatric outpatient clinic at Maharaj Nakorn Chiang Mai Hospital between 1 November 2024 and 30 September 2025. Research assistants not involved in clinical care invited eligible patients to participate after obtaining informed consent.

All participants completed an on-site questionnaire comprising: i) Demographic information; ii) The Thai version of the Rapid Mood Screener (RMS-T); and iii) Additional measures for convergent/divergent validity, including the Mood Disorder Questionnaire (MDQ) and Patient Health Questionnaire-9 (PHQ-9).

Diagnoses were confirmed by trained psychiatrists who were blinded to RMS-T/MDQ/PHQ-9 results using MINI and DSM-5-TR criteria. For test–retest reliability, a subset of participants randomly selected completed the RMS-T again after two weeks.

### Study design

The study of RMS-T comprised two phases: translation and cultural adaptation, and psychometric evaluation.

#### Translation and cultural adaptation.

The RMS was translated into Thai (RMS-T) following the World Health Organization (WHO) guidelines and Brislin’s forward–backward translation method. Two bilingual psychiatrists independently translated the RMS into Thai, followed by reconciliation and back-translation by two independent translators. A pre-final version was field-tested with 15 participants (3 with BD and 12 with MDD) to provide clarity and cultural appropriateness before finalization.

Cultural adaptation was performed by an expert committee consisting of psychiatrists and bilingual professionals. The committee reviewed each item for semantic, idiomatic, experiential, and conceptual equivalence. Discrepancies between the two forward translations mainly involved wording choices, levels of formality, and the nuances of psychiatric terminology. These discrepancies were discussed by the translators, and an expert panel consensus was achieved by selecting the wording that most accurately reflected the conceptual meaning of the original item while remaining clear and culturally appropriate for Thai respondents. For example, the phrase “unusually energetic” was carefully reviewed because a direct Thai translation could be interpreted as simply having much power in doing physical activities. The final wording was refined to better reflect a noticeable increase in energy beyond an individual’s usual level, consistent with the clinical features of hypomania or mania.

#### Psychometric evaluation.

Psychometric evaluation included internal consistency, test–retest reliability, and validity. The RMS-T was administered to 140 participants (31 with BD and 109 with MDD) during outpatient visits. A subset of participants (n = 30; ≈ 20%, including 24 with MDD and 6 with BD) selected through simple random sampling, completed the RMS-T again after two weeks to assess test–retest reliability.

### Measurement

1) Rapid Mood Screener (RMS-T): A six-item self-report tool assessing BD risk factors and manic symptoms.
2) The Thai Mood Disorder Questionnaire (T-MDQ): A validated Thai version was used for convergent validity. It was found to have a Cronbach’s alpha of 0.791, sensitivity of 0.77, and specificity of 0.73 at a cutoff of 5 in discriminating between MDD and BD [21]. The MDQ is composed of three parts. In part one, the MDQ screens for a lifetime history of manic or hypomanic symptoms using 13 yes/no items. The second part asks whether several manic or hypomanic symptoms have been experienced during the same period. Part three assesses the functional impairment due to the illness on a 4-point scale (“no” to “severe”).
3) Patient Health Questionnaire-9 (PHQ-9): Thai version for depressive symptom severity was translated by Lotrakul M, et al, 2008. The optimal cutoff score of Thai PHQ-9 ≥ 9 revealed a sensitivity of 84%, a specificity of 77% [22].
4) MINI: Structured diagnostic interview used as the gold standard for BD and MDD diagnosis. The Thai version 5.0.0-Revised 2007 was administered. The kappa and sensitivity on the diagnosis of current major depressive episode, current suicide risk, lifetime psychotic disorder, manic episode, and current generalized anxiety disorder were very high (> 0.75, > 0.81, respectively). The specificity, the negative predictive value, and efficiency were very high on every diagnosis (> 0.81) [23].

### Study procedures

Participants completed the RMS-T, T-MDQ, and Thai PHQ-9 during their clinical visits. Completion time and feedback on language clarity were recorded. For test–retest reliability, participants were contacted via email or text message and provided with an online RMS-T link (Google Form) two weeks after the initial assessment, with one reminder call.

### Statistical analysis

Internal consistency was assessed using Cronbach’s alpha and McDonald’s omega. Although an alpha or omega larger than 0.7 is widely accepted, this study set an alpha or omega larger than 0.6 as acceptable, given the RMS-T’s brevity (6 items) and the inclusion of heterogeneous clinical indicators (e.g., manic symptoms vs. age of onset) [24,25].

Test–retest reliability was measured using intraclass correlation coefficient (ICC). Criterion validity was examined by comparing RMS-T scores with MINI diagnoses and MDQ, using receiver operating characteristic (ROC) curve analysis. Area under the curve (AUC) values were calculated for BD, BD-I, and BD-II. Because the optimal RMS cutoff is ≥ 4, we evaluated RMS-T cutoffs at ≥ 3, ≥ 4, and ≥ 5. The optimal cutoffs were identified using Youden’s index. Analyses were performed using Jamovi version 2.6, with significance set at p < 0.05.

## Results

### Participant characteristics

A total of 140 participants were enrolled, including 31 with BD (BD-I = 21, BD-II = 10) and 109 with MDD. All MINI-confirmed diagnoses were consistent with the diagnoses from the clinical interviews by psychiatrists, according to DSM-5-TR. The mean age was 29.0 years (SD 10.9), and 77.1% were female. Depressive symptoms assessed by PHQ-9 were highest in BD-I, followed by MDD and BD-II. RMS-T and MDQ scores were significantly higher in the BD groups compared with MDD (Table 1).

**Table 1:** Demographic characteristics of participants.

Variables total	MDD (%) n = 109	BD (%) n = 31	BD I (%) n = 21	BD II (%) n = 10	Total n (%) n = 140	P-value^a^
Age (years), mean (SD)	29.07 (11.31)	28.68 (9.34)	29.71 (10.25)	26.50 (7.04)	29.0 (10.9)	0.501
Sex						0.967
Male	25 (22.9)	7 (22.6)	6 (28.6)	1 (10.0)	32 (22.9)	
Female	84 (77.1)	24 (77.4)	15 (71.4)	9 (90.0)	108 (77.1)	
Education level						0.859
Primary school	1 (0.9)	0 (0.0)	0 (0.0)	0 (0.0)	1 (0.7)	
Secondary school	13 (11.9)	4 (12.9)	3 (14.3)	1 (10.0)	17 (12.1)	
Post-secondary school	95 (87.2)	27 (87.1)	18 (85.7)	9 (90.0)	122 (87.1)	
Marital status						0.342
Single	84 (77.1)	27 (87.1)	18 (85.7)	9 (90.0)	111 (79.3)	
Married	20 (18.3)	4 (12.9)	3 (14.3)	1 (10.0)	24 (17.1)	
Divorced/Widowed	5 (4.6)	0 (0.0)	0 (0.0)	0 (0.0)	5 (3.6)	
Underlying disease						0.008
Yes	71 (65.1)	12 (38.7)	7 (33.3)	5 (50.0)	83 (59.3)	
No	38 (34.9)	19 (61.3)	14 (66.7)	5 (50.0)	57 (40.7)	
Family History of Mood Disorder						0.336
Yes	20 (18.3)	8 (25.8)	7 (33.3)	1 (10.0)	28 (20.0)	
No	89 (81.7)	23 (74.2)	14 (66.7)	9 (90.0)	112 (80.0)	
Income per month						0.229
<10,000 THB (<$300)	46 (42.2)	9 (29.0)	5 (23.8)	4 (40.0)	55 (39.3)	
10,001–20,000 THB ($300 – $600)	35 (32.1)	15 (48.4)	11 (52.4)	4 (40.0)	50 (35.7)	
>20,000 THB (>$600)	28 (25.7)	7 (22.6)	5 (23.8)	2 (20.0)	35 (25.0)	
Financial Stress (Hospital bills)						0.126
Not worried	52 (47.7)	15 (48.4)	9 (42.9)	6 (60.0)	67 (47.9)	
Slightly worried	44 (40.4)	8 (25.8)	5 (23.8)	3 (30.0)	52 (37.1)	
Worried	9 (8.3)	7 (22.6)	6 (28.6)	1 (10.0)	16 (11.3)	
Very worried	4 (3.7)	1 (3.2)	1 (4.8)	0 (0.0)	5 (3.5)	
PHQ-9 scores, mean (SD)	17.17 (5.66)	17.13 (7.21)	17.95 (7.05)	15.40 (7.60)	17.20 (6.01)	0.708
RMS-T scores, mean (SD)	2.24 (1.50)	4.03 (1.38)	3.95 (1.47)	4.20 (1.23)	2.64 (1.65)	**<0.001**
T-MDQ scores, mean (SD)	4.11 (2.75)	8.39 (3.44)	8.33 (3.12)	8.50 (4.22)	5.06 (3.41)	**<0.001**

**Abbreviations:** MDD, Major Depressive Disorder; BD, Bipolar disorder; SD, standard deviation; PHQ-9, Patient Health Questionnaire-9; RMS-T, The Rapid Mood Screener-Thai version; T-MDQ, Thai Mood Disorders Questionnaire.

^**a**^the p-values < 0.05. Chi-squared tests were used for categorical variables, and t-tests or Mann-Whitney U tests were used for continuous data.

### Reliability and item analysis

Internal consistency was acceptable for a brief tool (Cronbach’s alpha = 0.610; McDonald’s omega = 0.632). All six items correlated significantly with the total score, and item-total correlations ranged from 0.356 to 0.748 (Table 2). Despite high item-total correlations for specific items (e.g., Item 5, r = 0.748), the overall alpha remains modest, reflecting the brevity of the screener, which prioritizes clinical breadth and practicality over item redundancy. An if-item-deleted analysis of Cronbach’s alpha was performed. Deleting item 1 improved Cronbach’s alpha to 0.653 and McDonald’s omega to 0.671, representing a modest increase, but reliability remained below 0.70, and the overall interpretation was unchanged.

**Table 2:** Assessment of Spearman’s correlation and P-value of RMS-T and its items.

	Item 1	Item 2	Item 3	Item 4	Item 5	Item 6
r with total RMS-T score	0.356	0.561	0.471	0.661	0.748	0.627
p-value^**a**^	<0.001	<0.001	<0.001	<0.001	<0.001	<0.001

**Abbreviations:** RMS-T, Rapid Mood Screener-Thai version.

^**a**^*p <* 0.01; *r* is correlation coefficient.

Correlation between RMS-T and T-MDQ was moderate (Spearman’s rho = 0.551, p < 0.001; 95% CI: 0.457–0.680). Test–retest reliability in 30 participants was strong (ICC = 0.887; 95% CI: 0.774–0.944).

### Exploratory factor analysis

The overall Kaiser–Meyer–Olkin Measure of Sampling Adequacy (KMO) measure was 0.628, and Bartlett’s test was significant, χ²(15) = 122.0, p < 0.001, supporting factorability of the correlation matrix. Exploratory factor analysis (EFA) using minimum residual extraction yielded a one-factor solution, accounting for the common variance among the six items. Although the original RMS was conceptualized as multidimensional, only one factor demonstrated an eigenvalue greater than one in the present sample. Item 1 showed a low measure of sampling adequacy (MSA = 0.407) and high uniqueness (0.989), suggesting limited contribution to the extracted factor (Table 3).

**Table 3:** Exploratory Factor Analysis of the RMS-T.

Item	Factor Loading	Uniqueness
Item 1	<0.100	0.989
Item 2	0.317	0.900
Item 3	0.370	0.863
Item 4	0.645	0.584
Item 5	0.840	0.295
Item 6	0.455	0.793

**Abbreviations:** RMS-T, Rapid Mood Screener-Thai version.

Because item 1 demonstrated a low measure of sampling adequacy and high uniqueness, a sensitivity analysis was performed excluding this item. The resulting EFA continued to support a one-factor solution, with improved sampling adequacy (KMO = 0.660) and all remaining items demonstrating acceptable MSA values (>0.60).

### Criterion validity

**Fig 1:**
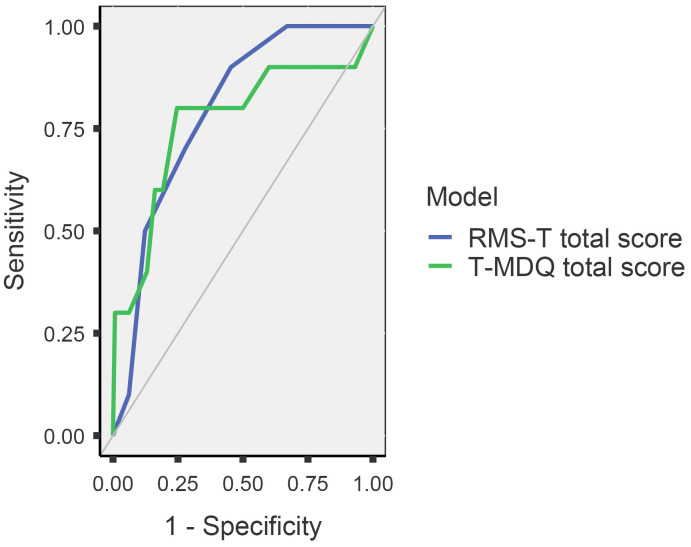
The ROC curves of the RMS-T and T-MDQ in predicting patients with BD. **Abbreviation:** ROC, Receiver operating characteristic; RMS-T, Rapid Mood Screener-Thai version; T-MDQ, Thai Mood Disorder Questionnaire.

**Fig 2:**
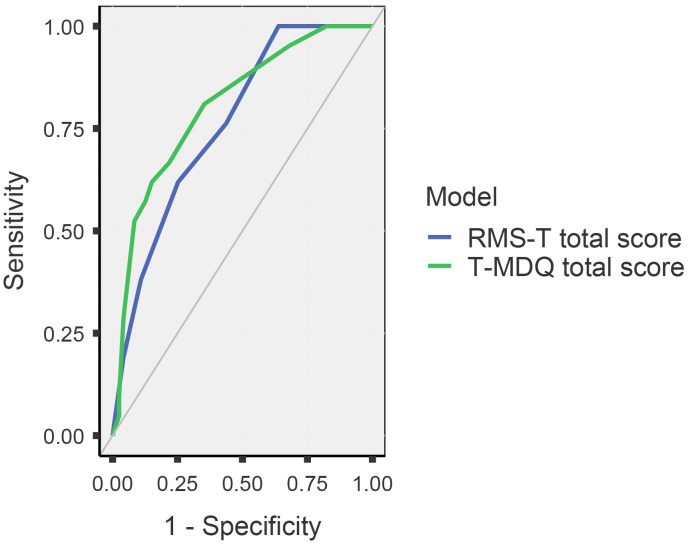
The ROC curves of the RMS-T and T-MDQ in predicting patients with BD-I. **Abbreviation:** ROC, Receiver operating characteristic; RMS-T, Rapid Mood Screener-Thai version; T-MDQ, Thai Mood Disorder Questionnaire.

**Fig 3:**
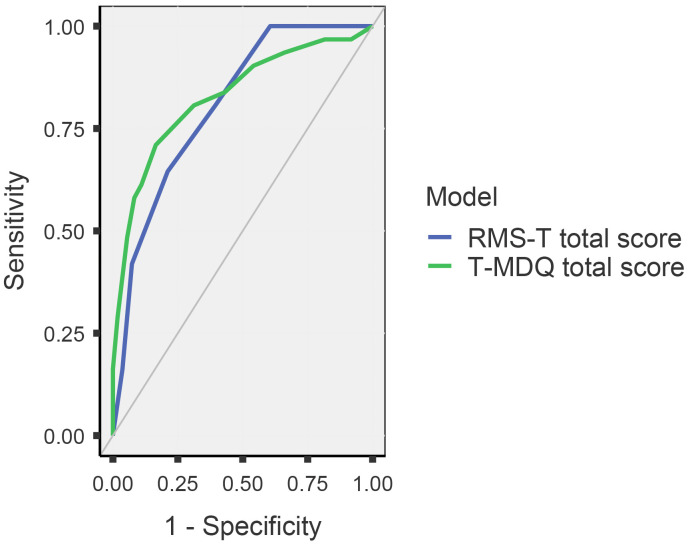
The ROC curves of the RMS-T and T-MDQ in predicting patients with BD-II. **Abbreviation:** ROC, Receiver operating characteristic; RMS-T, Rapid Mood Screener-Thai version; T-MDQ, Thai Mood Disorder Questionnaire.

ROC analysis showed good discriminative ability of RMS-T for BD (AUC = 0.805; 95% CI: 0.728–0.882), BD-I (AUC = 0.761; 95% CI: 0.664–0.859), and BD-II (AUC = 0.791; 95% CI: 0.675–0.906). Performance was comparable to T-MDQ (AUC = 0.828; 95% CI: 0.737–0.919 for BD; AUC = 0.806 for BD-I) (Figs 1–3). Differences in AUC between RMS-T and T-MDQ were not statistically significant (AUC difference for BD: −0.0229; 95% CI: −0.106–0.0600; p = 0.588, BD-I: −0.0446; 95% CI: −0.142–0.0530; p = 0.370, BD-II: 0.0262; 95% CI: (−0.0899–0.142; p = 0.659).

### Optimal cutoffs

For BD, the optimal RMS-T cutoff was ≥ 4 items (sensitivity = 64.52%, 95% CI: 0.454–0.807; specificity = 78.90%, 95% CI: 0.700–0.861; PPV 46.51%, 95% CI: 0.357–0.576; NPV 88.66%, 95% CI: 0.828–0.927; Youden’s index = 0.43). For BD-I, the same cutoff provided the best balance (sensitivity 61.90%, 95% CI: 0.384–0.819; specificity 74.79%, 95% CI: 0.660–0.823; PPV 30.23%, 95% CI: 0.215–0.406; NPV 91.75%, 95% CI: 0.865–0.951; Youden’s index 0.37) (Table 4).

**Table 4:** RMS-T test performance by optimal cutoff selection.

Cutoff point	Total BD (n = 31)					BD-I (n = 21)				
	Sensitivity (%)	Specificity (%)	PPV (%)	NPV (%)	Youden Index	Sensitivity (%)	Specificity (%)	PPV (%)	NPV (%)	Youden Index
3	80.65	60.55	36.76	91.67	0.41	76.19	56.30	23.53	93.06	0.33
**4**	**64.52**	**78.90**	**46.51**	**88.66**	**0.43**	**61.90**	**74.79**	**30.23**	**91.75**	**0.37**
5	41.94	92.66	61.90	84.87	0.35	38.10	89.08	38.10	89.08	0.27

**Abbreviations:** NPV, negative predictive value; PPV, positive predictive value.

Due to the limited number of 10 participants, the BD-II subgroup analysis should be considered exploratory. For BD-II, the optimal cutoff was ≥ 3 items (sensitivity 90.00%, 95% CI: 0.555–0.998; specificity 54.62%, 95% CI: 0.457–0.634; PPV 13.24%, 95% CI: 0.103–0.168; NPV 98.61%, 95% CI: 0.917–0.998; Youden’s index 0.45).

## Discussion

The present study confirms that the Thai version of the Rapid Mood Screener (RMS-T) is a reliable and valid screening tool for bipolar disorder (BD) among patients presenting with depressive symptoms. RMS-T demonstrated acceptable internal consistency, robust test–retest reliability, and satisfactory discriminative ability for BD, comparable to the Thai Mood Disorder Questionnaire (T-MDQ). Optimal cutoff thresholds were determined as ≥ 4 for BD and BD-I, providing an appropriate balance between sensitivity and specificity.

The RMS-T results in this study align with previous findings on reliability and validation. The present Cronbach’s alpha of 0.610 is comparable to the Chinese version (0.66) [18]. However, this value is lower than that of the T-MDQ (0.791), likely due to the brevity of the RMS-T, which comprises only six items [26,27]. Additionally, RMS-T includes heterogeneous items assessing manic symptoms and historical clinical features associated with bipolar disorder, rather than measuring a single homogeneous construct [28]. Therefore, an alpha above 0.60 is considered acceptable [29] for brief screening tools with clinically diverse indicators [30]. Several items demonstrated modest factor loadings. This may be attributable to the heterogeneous content of the RMS, which combines symptom-based indicators with illness-course characteristics. Such heterogeneity may reduce inter-item correlations while preserving the instrument’s clinical utility as a screening tool. Nonetheless, exploratory factor analysis supported a single-factor structure for the Thai version of the RMS. Although the RMS includes items assessing both manic symptoms and bipolar illness-course characteristics, the present findings suggest that these items may collectively reflect a broader underlying construct of bipolarity risk in Thai patients. The original RMS was developed primarily as a screening instrument rather than as a scale with established latent dimensions. Therefore, the emergence of a single-factor solution in the present study does not necessarily contradict the conceptual framework of the original instrument. The RMS-T cutoff of four for distinguishing BD from MDD is also identical to the original RMS [16].

A sensitivity analysis excluding item 1 yielded similar findings and did not alter the overall one-factor structure. Although removal of item 1 improved the overall KMO value and item-level sampling adequacy, the factor solution remained unchanged. These findings suggest that item 1 may be less strongly associated with the underlying bipolarity factor measured by the RMS-T, while having minimal impact on the overall dimensionality of the instrument.

Meta-analytic evidence suggests that RMS generally performs well compared to other screening tools for BD detection, with a pooled sensitivity and specificity of 0.78 and 0.72, respectively [31]. However, diagnostic accuracy varies by subtype: RMS shows strong performance for BD-I but is less robust for BD-II, where instruments like the MDQ or Bipolarity Index (BI) may perform better [31]. This aligns with our observation that RMS-T, while effective overall, was less sensitive for BD-II, although the small sample size of BD-II participants largely limits its interpretation, and the cutoff should be considered exploratory.

The sensitivity of the RMS-T in the present study (64.5%) was lower than that reported in the original validation study (88%). Although this discrepancy suggests that contextual or cultural factors may influence the performance of self-administered screening tools, the present study did not include direct assessments, such as item-level response pattern analyses or stigma measures, to empirically evaluate this hypothesis. Moreover, our pilot testing during adaptation focused on item clarity rather than endorsement patterns.

Existing literature on the Thai context has suggested that sociocultural factors, including stigma toward psychiatric illness and norms emphasizing social harmony and family reputation, may influence symptom disclosure [32–34]. However, in the absence of direct supporting data, these factors should be interpreted as plausible, but untested, explanatory considerations rather than causal mechanisms. Variability across studies may reflect cultural differences in symptom expression and interpretation [35]. Despite these challenges, RMS has shown consistent validity across diverse settings, including the United States and China [16,18].

Recall bias and limited illness insight could further reduce accurate reporting of past manic and hypomanic episodes. Additionally, in contrast to the original RMS validation, which primarily focused on bipolar I disorder, the present study included both bipolar I and bipolar II disorder. Hypomanic symptoms characteristic of bipolar II disorder are typically subtler, episodic, and often perceived as ego-syntonic, which may lead to under-recognition and lower endorsement in self-report screening instruments. Lastly, differences in diagnostic instruments may also contribute to outcome variation.

Previous research has also noted differences in MDQ performance among Thai participants compared with Western populations [21]. While T-MDQ demonstrated slightly higher sensitivity than RMS-T in our sample, RMS-T offers advantages in brevity (6 vs. 13 items) and the inclusion of BD risk factors, making it more practical for non-specialist settings, especially in low-resource countries such as Thailand.

Beyond diagnostic accuracy, the use of RMS has been associated with substantial cost savings in healthcare. A recent economic model from the United States estimated that screening patients with depressive symptoms using RMS could save approximately $1,279 per patient in the first year and over $3,000 cumulatively within three years by reducing misdiagnosis of bipolar I disorder and associated inappropriate treatments [17]. These savings were robust even under conservative assumptions, such as lower prevalence rates and reduced sensitivity. This highlights that even modest improvements in diagnostic accuracy can translate into meaningful reductions in healthcare resource utilization and costs, and the same cost‑effectiveness analysis should be replicated in Thai settings.

For clinical practice in Thailand, RMS-T offers a culturally adapted, time-efficient tool (average completion time under two minutes) with a distinct item structure that facilitates earlier identification of BD. Its integration into psychiatric and primary care settings can help prevent inappropriate antidepressant monotherapy, improve treatment outcomes, and optimize resource allocation. By combining brevity with clinically relevant risk factors, RMS-T represents a pragmatic approach to improving diagnostic precision and supporting better patient care, which can be similarly adopted in other countries with similar mental health resource shortages.

Future studies should explore the utility of RMS-T in primary care and community settings, assess cost-effectiveness, and evaluate its predictive validity for long-term outcomes. Comparative studies with other screening tools in diverse Thai populations would further strengthen evidence for its clinical adoption. Furthermore, as Item 1 demonstrated low sampling adequacy and extremely high uniqueness, it contributed little to the extracted factor. This finding may reflect cultural, linguistic, or clinical differences affecting the interpretation of this item in the Thai context. Further studies are needed to qualitatively determine whether this represents a translation issue or a genuine difference in symptom expression.

This study has several limitations. Firstly, it was conducted in a hospital-based sample at a psychiatric outpatient unit, which may restrict the generalizability of findings to the lower-prevalence primary care settings or broader community, and does not fully represent the heterogeneity of individuals with and without mood disorders. Secondly, although the Thai version of the MINI has demonstrated good validity, its diagnostic accuracy for hypomanic episodes is moderate, as reflected by lower kappa values, which may have influenced case classification [36]. Given its modest internal consistency, RMS-T results should be interpreted cautiously and in conjunction with clinical judgment. Thirdly, the relatively small number of participants with BD-II limits the precision of sensitivity estimates and should be considered preliminary and underpowered, warranting BD‑II‑focused research. In addition, the sample size of 140 was adequate and exceeded commonly recommended subject-to-item ratios for exploratory factor analysis of brief instruments. However, the relatively small number of items may limit the stability of the extracted factor structure. Confirmatory factor analysis in larger and more diverse samples is warranted to further evaluate the dimensionality of the RMS-T. Lastly, the cross-sectional design precludes evaluation of predictive validity; therefore, it remains unclear whether individuals with MDD and high RMS-T scores will later transition to BD.

## Conclusion

The RMS-T is a time-efficient and clinically practical screening tool for BD in Thai patients with depressive symptoms to improve diagnostic accuracy and treatment outcomes potentially. Its moderate psychometric properties support its integration into routine psychiatric assessment as an initial assessment, followed by a complete clinical evaluation for bipolar disorder among those who screen positive. Further population-based studies would ensure its cost-effectiveness and usefulness in other clinical settings.

## Supporting information

S1 File The translation of RMS. (DOCX)
