# Supplementary material for: Reliability and validity of the Thai version of the Rapid Mood Screener (RMS-T)
Source: PLoS One. 2026 Aug 6;21(8):e0355733. doi: 10.1371/journal.pone.0355733 (PMC13446682; doi:10.1371/journal.pone.0355733)
Supplement: Supplementary file 1 [file pone.0355733.s001.docx]

**Rapid Mood Screener**

| **Item** | **Response** | |
| --- | --- | --- |
| 1. Have there been at least 6 different periods of  time (at least 2 weeks) when you felt  deeply depressed? | Yes | No |
| 2. Did you have problems with depression before  the age of 18? | Yes | No |
| 3. Have you ever had to stop or change your  antidepressant because it made you highly  irritable or hyper? | Yes | No |
| 4. Have you ever had a period of at least 1 week  during which you were more talkative than  normal with thoughts racing in your head? | Yes | No |
| 5. Have you ever had a period of at least 1 week  during which you felt any of the following:  unusually happy; unusually outgoing; or  unusually energetic? | Yes | No |
| 6. Have you ever had a period of at least 1 week  during which you needed much less sleep  than usual? | Yes | No |

**Rapid Mood Screener-Thai version**

| **รายการ** | **คำตอบ** | |
| --- | --- | --- |
| 1. คุณเคยมีช่วงเวลาที่รู้สึกซึมเศร้าอย่างมาก (ติดต่อกันนานอย่างน้อย 2 สัปดาห์) อย่างน้อย 6 ช่วงที่แตกต่างกันหรือไม่ | ใช่ | ไม่ใช่ |
| 2. คุณมีปัญหาเรื่องภาวะซึมเศร้าก่อนอายุ 18 ปีหรือไม่ | ใช่ | ไม่ใช่ |
| 3. คุณเคยต้องหยุดหรือเปลี่ยนยาต้านเศร้าเนื่องจากยาทำให้คุณรู้สึกหงุดหงิดมากหรือทำกิจกรรมมากกว่าปกติหรือไม่ | ใช่ | ไม่ใช่ |
| 4. คุณเคยมีช่วงเวลาอย่างน้อย 1 สัปดาห์ที่พูดมากกว่าปกติร่วมกับมีความคิดแล่นเร็วหรือไม่ | ใช่ | ไม่ใช่ |
| 5. คุณเคยมีช่วงเวลาอย่างน้อย 1 สัปดาห์ที่คุณมีอาการดังต่อไปนี้อย่างน้อย 1 ข้อหรือไม่: มีความสุขอย่างผิดปกติ; เข้าสังคมมากขึ้นอย่างผิดปกติ; หรือมีเรี่ยวแรงมากขึ้นอย่างผิดปกติ | ใช่ | ไม่ใช่ |
| 6. คุณเคยมีช่วงเวลาอย่างน้อย 1 สัปดาห์ที่คุณต้องการนอนน้อยกว่าปกติหรือไม่ | ใช่ | ไม่ใช่ |
